# There is no place for Tobacco Industry sponsorship in Continuing Professional Development

**DOI:** 10.4102/jcmsa.v2i1.101

**Published:** 2024-12-02

**Authors:** Leslie London, Mvuyiso Talatala, Bilkish Cassim

**Affiliations:** 1 Division of Public Health Medicine, School of Public Health, University of Cape Town, Cape Town, South Africa; 2 Department of Psychiatry, Faculty of Health Sciences, University of the Witwatersrand, Johannesburg, South Africa; 3 Chris Hani Baragwanath Academic Hospital, Johannesburg, South Africa; 4 Department of Geriatrics, University of KwaZulu-Natal, Durban, South Africa

**Keywords:** continuing professional development, tobacco, conflict of interest, policy, professional practice, industry

## Abstract

Continuing Professional Development (CPD) plays a key role in ensuring that health professionals maintain competence in their professional roles. The delivery of CPD requires provision of education and information that is independent of vested interests. Recent efforts by the Tobacco Industry to move into provision and sponsorship of CPD for doctors represents an ethical challenge and potentially contravenes South Africa’s obligations under the Framework Convention on Tobacco Control. The College of Medicine of South Africa (CMSA) has taken the position that there is no place for tobacco company sponsorship of CPD activities in light of its mandate to promote the highest degree of skill and efficiency in medical and dental practice and to advance the highest ethical standards and professional conduct in the profession. This position is in line with the CMSA’s position on sponsorship of its own CPD activities and we urge other health institutions to adopt a similar approach. The Health Professions Council of South Africa (HPCSA) should issue guidelines to CPD accreditors to exclude tobacco company-sponsored activities from HPCSA-accredited CPD. This is particularly important at this point while South Africa’s parliament is considering further legislation to control tobacco-related harms.

The Colleges of Medicine of South Africa (CMSA) has, as its mandate, the role of promoting the ‘highest degree of skill and efficiency in medical and dental practice’ and advancing ‘the highest ethical standards and professional conduct … not for pecuniary profit, but for the betterment of humanity’ (see https://cmsa.co.za/#:~:text=The%20CMSA%20is%20dedicated%20%E2%80%9CTo,for%20the%20betterment%20of%20humanity%E2%80%9D). In 2024, the CMSA adopted a policy prohibiting acceptance of donations or support from health-harming industries. Producing well-trained specialists and sub-specialists as national resources will make a key contribution to the South African health system. However, it is critical that the quality of training provided is constantly monitored and maintained. Through its assessment processes, which guide learning, the CMSA and its constituent colleges, strive to ensure best practice in examination.

At the same time, we recognise that after passing fellowship examinations, registering as a specialist or sub-specialist and entering the world of clinical practice, medical professionals must maintain their expertise through continuing education activities or Continuing Professional Development (CPD). This is both an ethical and professional obligation, which is currently managed through the CPD system of the Health Professions Council of South (HPCSA). The importance of CPD for maintenance of clinical skills has been widely acknowledged and institutionalised in health systems across the world^1,2^ with increasing attention to identifying the most effective methods of CPD to support enhanced quality of care and professional behaviour by clinicians.^3^ Indeed, conceptual models that locate CPD within the goals of population health improvement have attempted to broaden the mandate of such ongoing training.^4^

Who provides or sponsors CPD has been the subject of discussion in many contexts, principally because of the conflict of interest that arises when CPD is provided and/or sponsored by industries with a vested interest. Attention has been paid in public debates to the role of the pharmaceutical and other healthcare technology industries^5^ whose support for CPD has been found to be associated with increased prescribing of a sponsor’s product and increased sale of a sponsor’s drug through promotion of off-label uses.^6^

Efforts to manage conflict of interest related to pharmaceutical and healthcare product industry sponsorship (whether through external or self-regulation) have generally focused on ensuring that the contents, programme design, implementation and evaluation processes are free from commercial influence and that sponsors cannot influence the delivery of CPD.^7,8,9^

However, where industry’s products are potentially health harming, such as is the case with tobacco, alcohol, sugar-sweetened foods and beverages, the balancing of ‘commercial and public interests in prioritising biomedical, social and environmental aspects of health’^10^ is less complex when it comes to funding of professional societies’ activities. If one accepts, as many professional organisations currently do,^11,12^ that it is not ethical to accept contributions from donors seeking to influence their positions, or if the donation is not consistent with the society’s mission, then it should be clear that they should not accept sponsorship for CPD from companies whose profits are predicated on increased sales of commodities whose high levels of consumption adversely impact human and societal health.

In addition, among industries that market unhealthy products of no direct health benefits, the Tobacco Industry stands out being particularly problematic. Public disclosure of secret documents following litigation exposed the Tobacco Industry as having deliberately misled the public about health risks of tobacco products and the industry continues to manipulate science to promote its interest.^13,14,15,16^ The Tobacco Industry has also, for decades, facilitated the creation of supposedly independent front groups, to assist in covert public misinformation and to enable influence on policymakers who might otherwise be reluctant to work directly with industry.^16,17,18,19^ This strategy has gone hand in hand with efforts to co-opt the language of Harm Reduction,^20^ while still maximising sales, profit and shareholder return on all its products (both cigarettes and ENDS), thus nullifying any harm reduction intent, while manufacturing reputational benefits and access to policymakers.^21^

To reduce the massive burden associated with smoking and control the activities of the tobacco industry, the Framework Convention for Tobacco Control (FCTC)^22^ was adopted by the World Health Assembly in 2003, one of only two global conventions on health adopted by the United Nations General Assembly. South Africa was an early adopter of tobacco control measures for which the former Minister of Health, Nkosazana Dlamini-Zuma, received a Tobacco Free World award from the World Health Organization (WHO) in recognition of her ‘groundbreaking work’ in driving regulatory and programmatic measures for the reduction of tobacco consumption.^23^

Key measures required under the Convention include the obligation to ‘prohibit all forms of tobacco advertising, promotion and sponsorship’ and to ensure the provision of ‘effective and appropriate training … on tobacco control’. In that regard, the Convention Secretariat guidelines^24^ clearly state that:

[*M*]easures to ensure that entities involved in education, communication and training, and related research, including but not limited to academia, professional associations and governmental agencies, [*should*] fully respect the principles laid down in Article 5.3 of the Convention and its guidelines, and thus *do not accept any direct or indirect tobacco industry funding*. [*authors’ italicisation*]

The guidelines for implementing Article 5.3 explicitly call on governments to raise awareness about front groups acting openly or covertly ‘to further the interests of the tobacco industry’ and aim to protect against tobacco interference, either directly or via proxies.^25^

Yet, despite these clear invocations, Philip Morris International (PMI), one of the largest tobacco products companies in the world, has made it a priority to support continuing medical education efforts worldwide on tobacco harm reduction, arguing its strategy ‘is vital to improve public health’.^26^ In presenting itself as promoting Electronic Nicotine Delivery Systems (ENDS), PMI has simultaneously continued to invest in new cigarette brands, bought up new cigarette businesses, set up new manufacturing deals, challenged effective regulations and deliberately undermined the WHO FCTC.^27^ In the words of the Editor of the *Lancet* in 2019 ‘PMI continues to thrive on a global addiction to tobacco’^28^ and the company sold over 600 billion cigarettes in 2023 across the world.^29^

While it is true that ENDS reduce exposure to some tobacco-related toxicants, the WHO has publicly stated that ENDS products should be strictly regulated consistent with the provisions of the Convention as the major constituent of ENDS is nicotine, the highly addictive component of tobacco smoke.^30^ The role of ENDS in promoting cigarette use (i.e., as a gateway to tobacco use) is of enormous public health concern,^31^ notwithstanding industry denials of this risk (see https://exposetobacco.org/news/pmi-smoke-free-future/). The science of harm associated with ENDS is still being evaluated with significant evidence emerging regarding ENDS’ own risk profile being comparable to that of cigarettes.^32,33^ The International Agency for Research on Cancer, the specialised cancer agency of the WHO, recently announced that ENDS will be one of its priority exposures for assessment based on latest available scientific evidence. Until consensus is reached on the health risks associated with ENDS, ENDS must be treated as tobacco products in law. A recent WHO Technical Note confirms this position.^34^

The idea, therefore, that a tobacco company or a front for the tobacco industry, should sponsor continuing education on harm reduction when there remains considerable uncertainty about the efficacy and relative safety of ENDS, and when the company continues to aggressively market cigarettes in countries with weak legislation, is a conflict of interest that has been roundly condemned by experts in tobacco control globally.^35^ Dr Pamela Ling, director of the Center for Tobacco Control Research and Education at the University of California, San Francisco, in commenting on tobacco industry efforts to sponsor CPD activities, noticed that ‘the entry into the world of medical education is particularly audacious’ and that:

[*I*]n the past, medical education sponsored by the manufacturers of the leading preventable cause of death would have been ridiculous. As tobacco companies remake their image into pharmaceutical-like nicotine purveyors, it appears they have been emboldened to enter this arena.^36^

Why is this of relevance in South Africa today? The Tobacco Products and Electronic Delivery Systems Control Bill was introduced to the National Assembly by the Minister of Health in 2022 (see https://www.parliament.gov.za/bill/2307574), underwent public consultations in 2023 (see https://www.parliament.gov.za/press-releases/media-alert-committee-health-starts-public-hearings-tobacco-products-and-electronic-delivery-systems-control-bill) and a contested consultation process in 2024 (https://protectournext.co.za/tc-bill-public-consultations/ and https://www.news24.com/fin24/companies/nedlac-slams-tobacco-bill-consultation-calls-for-halt-to-parliamentary-process-20240906). It has now been reintroduced to the Portfolio Committee on Health on 04 September 2024 (https://pmg.org.za/committee-meeting/39419/). The Bill has been the subject of intense lobbying by industry to push back against its provisions (see, for example, https://www.atim.co.za/2023/09/21/press-release-citizens-voice-strong-support-for-tobacco-control-bill-countering-tobacco-industry-objections/ and https://www.news24.com/news24/politics/parliament/divergent-views-on-bill-to-stub-out-smoking-in-public-20231128). Philip Morris International, which markets both cigarettes and ENDS products, has actively lobbied against the Bill because it will bring ENDS and cigarettes under a single legislative framework, enabling regulations specific to different products. Interestingly, part of Industry’s push back in South Africa against the bill has been framed as willingness to see ENDS regulated as pharmaceutical rather than as a tobacco product, a strategy that industry has pursued elsewhere to ensure its survival in an increasingly regulated environment that threatens its profitability.

In the period in which the Bill was the subject of public hearings, PMI sought to sponsor CPD talks through local South African Independent Provider Associations. As pointed out earlier in the text, talks on harm reduction should not be provided by consultants working for the tobacco industry or its front groups, nor should the tobacco industry have any influence over the CPD programmes provided for medical practitioners. This is particularly so given the Convention’s insistence in Article 5.3 that public awareness should be raised about the use of front groups to advance the tobacco industry’s interests and the research evidence that the article’s application has proven successful in limiting industry’s interference with tobacco control measures.^16^ We cannot be sure whether the CPD offering was an attempt to influence policy. However, it is the case that one of the consultants involved in PMI-sponsored CPD was also an active participant in public hearings on the Bill in one of the provinces during parliamentary outreach. As has been argued elsewhere, the Tobacco Industry has no business funding Continuing Medical Education (CME), and such practices would be particularly egregious if this were intended to influence legislative processes.^37^

The CMSA was a signatory to a complaint directed to the HPCSA earlier this year concerning tobacco industry sponsorship of CPD activities. The CMSA did so because it has adopted its own policy on Sponsorship and Advertising, which very clearly precludes acceptance of funding from entities whose mission is contrary to the goals of the CMSA and whose funding may serve to whitewash or greenwash corporate interests or aim to influence health policy and the practice of future specialists in ways that are antithetical to advancing healthcare and population health.

We believe that CPD is an essential complement to the training that the CMSA certifies through its examination processes as valid and fair reflections of practitioner competence. For that reason, CPD must remain independent of entities that have conflicts of interest. In particular, there is no place for tobacco company sponsorship of CPD activities. We urge other professional bodies to adopt similar positions which we believe are appropriate to our ethical and professional obligations as healthcare practitioners. In particular, we alert CPD accreditation committees and other registered accreditors regarding the importance of ensuring that CPD is free of vested interests. Accreditation committees are ‘independent’ bodies but take direction from the HPCSA as to what counts as credible CPD and have obligations to ensure the highest ethical standards are maintained when considering CPD applications.
